# A Case of Cholestatic Liver Involvement Secondary to Amyloid Light Chain Amyloidosis With New-Onset Hypercholesterolemia and Elevated Gamma-Glutamyltransferase Level

**DOI:** 10.7759/cureus.44001

**Published:** 2023-08-23

**Authors:** Yuta Nakano, Ryosuke Kawamoto, Eisaku Ito, Kayoko Matukawa

**Affiliations:** 1 Department of Nephrology, Ome Municipal General Hospital, Tokyo, JPN; 2 Department of Nephrology, Tokyo Medical and Dental University, Tokyo, JPN; 3 Department of Pathology, Ome Municipal General Hospital, Tokyo, JPN

**Keywords:** gamma glutamyltransferase, hypercholesterolemia, nephrotic syndrome, cholestatic liver involvement, al amyloidosis

## Abstract

Amyloid light chain (AL) amyloidosis is a rare disorder caused by the deposit of misfolded light chain proteins. AL amyloidosis causes multiple organ involvement and rarely causes fatal liver failure. We present a 68-year-old man who showed cholestatic liver injury and was diagnosed with AL amyloidosis. Due to rapidly progressing cholestatic liver involvement, the patient died five days after the renal biopsy. Preclinically, there was hypercholesterolemia, and levels of gamma-glutamyltransferase (GGT) were elevated. Previous studies have suggested hypercholesterolemia and elevated GGT levels in patients with AL amyloidosis and liver involvement; however, its clinical relevance remains unknown. Our report suggests that in addition to serum kappa/lambda, the combination of new-onset GGT level elevation and hypercholesterolemia could be preclinical characteristics of cholestatic liver involvement in AL amyloidosis.

## Introduction

Amyloidoses are rare disorders caused by the deposition of misfolded proteins [1]. Abnormal and β-sheet enriched proteins circulate in blood and affect multiple organs [2]. Among over 32 proteins known to cause amyloidosis, the monoclonal immunoglobulin light chain causes amyloid light chain (AL) amyloidosis, which is the most common form of systemic amyloidosis [1,2]. Systemic AL amyloidosis may affect multiple organs, including the heart, liver, kidneys, soft tissues, thyroid, and nervous system [2].

A previous study suggested that elevated levels of alkaline phosphatase (ALP) and hepatomegaly are hepatic manifestations caused by AL amyloidosis [2], with elevated total bilirubin levels indicating a worse prognosis [3]. Although liver involvement does not cause serious disease, fatal cholestatic liver failure rarely occurs [4]. Herein, we present the case of a patient with rapidly progressive multiorgan dysfunction with cholestatic liver involvement due to AL amyloidosis who presented new-onset hypercholesterolemia and elevated gamma-glutamyltransferase (GGT) levels in the preclinical stage.

## Case presentation

**Figure 1:**
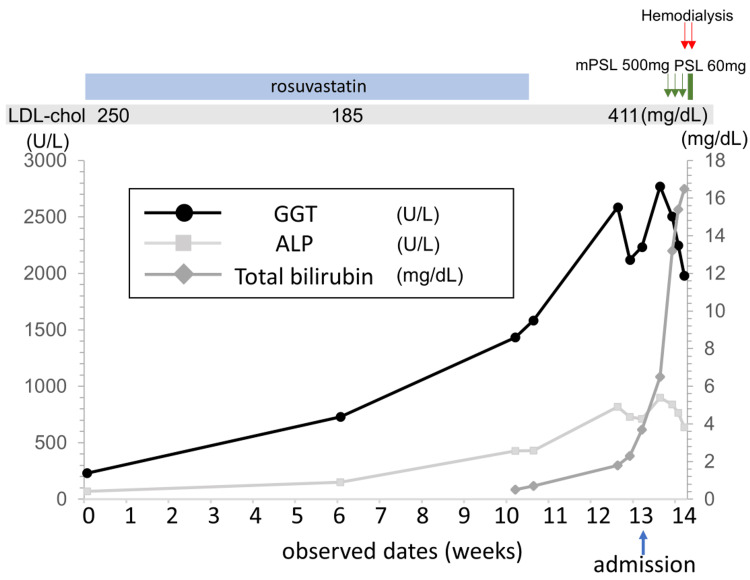
Clinical course There was an increase in GGT and LDL-cholesterol levels first, followed by ALP and total bilirubin levels. LDL-chol, low-density lipoprotein cholesterol, mPSL, methylprednisolone, PSL, prednisolone, GGT, gamma-glutamyltransferase, ALP, alkaline phosphatase

A 68-year-old Japanese man was admitted to our hospital because of liver injury, hypercholesterolemia, and hypoalbuminemia. Four months before admission, his low-density lipoprotein (LDL) cholesterol and gamma-glutamyltransferase levels were elevated despite being normal six months before. Simultaneously, his serum albumin level, renal function, and other liver enzymes were within the normal range. Hence, rosuvastatin was initiated for hypercholesterolemia (Figure 1, Table 1). However, follow-up tests showed that his alkaline phosphatase (ALP) levels were also elevated (Figure 1). His doctor suspected that the cause of liver injury was rosuvastatin, prompting its discontinuation. Nevertheless, his liver enzyme levels continued to increase, prompting referral to our hospital. On admission, he had bilateral lower limb edema, ascites, and pleural effusion but no dyspnea. There were no neurological deficits. Aside from rosuvastatin, he did not receive any medications, and his medical history was unremarkable. He had no history of alcohol abuse. His laboratory workup showed kidney injury, massive proteinuria, low serum albumin, hypercholesterolemia, and hypothyroidism, in addition to liver injury (Table 2). Antimitochondria antibody (AMA) and antinuclear antibody were negative. Hepatitis B virus antigen and C virus antibody titers were unreactive. Urinary Bence Jones protein was negative. Because of ascites and effusion, a liver biopsy was not performed. Cardiac ultrasound showed symmetric left ventricular hypertrophy (interventricular septum thickness: 14 mm, left ventricular posterior wall thickness: 13.1 mm) with normal ejection fraction (70%) measured by the Simpson method and no valve abnormalities. His electrocardiogram was within the normal range. As kidney function declined and low serum albumin was progressive, corticosteroid therapy was initiated to treat nephrotic syndrome. However, due to the progressive decrease in urine volume and increasing serum creatine level, hemodialysis was initiated.

**Table 1:** Trends of laboratory tests WBC, white blood cells; Hb, hemoglobin; Plt, platelet; CRP, C-reactive protein; TP, total protein; Alb, albumin; Cre, creatinine; Total Chol, total cholesterol; LDL-Chol, low-density lipoprotein cholesterol; HDL-Chol, high-density lipoprotein cholesterol; AST, aspartate aminotransferase; ALT, alanine aminotransferase

Weeks	-33	0	6	10	13(admission)
WBC	4800 /µL	4600 /µL	4700 /µL	4900 /µL	5950 /µL
Hb	12.8 g/dL	13.9 g/dL	14.3 g/dL	15.2 g/dL	16.4 g/dL
Plt	28.8×10^4^ /µL	35.6×10^4^ /µL	31.6×10^4^ /µL	33.1×10^4^ /µL	31.8×10^4^ /µL
CRP	0.04 mg/dL	N/A	N/A	0.32 mg/dL	0.78 mg/dL
TP	6.9 mg/dL	6.8 mg/dL	6.4 mg/dL	5.7 mg/dL	3.9 mg/dL
Alb	4.4 mg/dL	4.2 mg/dL	3.8 mg/dL	2.6 mg/dL	1.8 mg/dL
Cre	0.92 mg/dL	0.93 mg/dL	0.89 mg/dL	0.94 mg/dL	1.12 mg/dL
Total Chol	223 mg/dL	360 mg/dL	298 mg/dL	310 mg/dL	574 mg/dL
LDL-Chol	133 mg/dL	250 mg/dL	185 mg/dL	179 mg/dL	412 mg/dL
HDL-Chol	70 mg/dL	65 mg/dL	77 mg/dL	97 mg/dL	58 mg/dL
T-bill	N/A	N/A	N/A	0.5 mg/dL	2.3 mg/dL
D-bill	N/A	N/A	N/A	0.2 mg/dL	1.7 mg/dL
ASL	16 U/L	30 U/L	38 U/L	73 U/L	109 U/L
ALT	17 U/L	23 U/L	34 U/L	46 U/L	58 U/L

**Table 2:** Results of laboratory tests WBC, white blood cells; Hb, hemoglobin; Plt, platelet; CRP, C-reactive protein; TP, total protein; Alb, albumin; Cre, creatinine; Total Chol, total cholesterol; LDL-Chol, low-density lipoprotein cholesterol; HDL-Chol, high-density lipoprotein cholesterol; AST, aspartate aminotransferase; ALT, alanine aminotransferase; ALP, alkaline phosphatase; GGT, γ-glutamyltranspeptidase; HbA1c, hemoglobin A1c; IgG, immunoglobulin G; IgA, immunoglobulin A; IgM, immunoglobulin M; ANA, antinuclear antibody; AMA, antimitochondrial antibody; M-protein, myeloma protein; BNP, brain natriuretic hormone; TSH, thyroid-stimulating hormone; FT3, free triiodothyronine; FT4, free thyroxine; TPOAb, antithyroid peroxidase antibody; TgAb, antithyroglobulin antibody; Tg, thyroglobulin; TRAb, antithyroid-stimulating hormone receptor antibody; HBs antigen, hepatitis B surface antigen; HCV antibody, hepatitis C virus antibody; urinary RBC, urinary red blood cell; BJP, Bence Jones protein

	Patient values	Reference	Interpretation	
Blood test				
WBC	5950/µL	3300–8600	normal	
Hb	16.4 g/dL	13.7–16.8 g/dL	normal	
Plt	31.8 × 10^4^/µL	15.8 × 10^4^–34.8 × 10^4^/µL	normal	
CRP	0.78 mg/dL	0–0.3 mg/dL	normal	
TP	3.9 mg/dL	6.7–8.3 mg/dL	decreased	
Alb	1.8 mg/dL	4–5 mg/dL	decreased	
Cre	1.12 mg/dL	0.61–1.04 mg/dL	elevated	
Total Chol	574 mg/dL	150–219 mg/dL	elevated	
LDL-Chol	412 mg/dL	70–139 mg/dL	elevated	
HDL-Chol	58 mg/dL	40–80 mg/dL	normal	
Total bilirubin	2.3 mg/dL	0.4–1.5 mg/dL	elevated	
Direct bilirubin	1.7 mg/dL	<0.4 mg/dL	elevated	
ASL	109 U/L	13–39 U/L	elevated	
ALT	58 U/L	10–42 U/L	elevated	
ALP	728 U/L	38–113 U/L	elevated	
GGT	2122 U/L	13–64 U/L	elevated	
HbA1c	6.0%	4.9%–6.0%	normal	
IgG	343 mg/dL	861–1747 mg/dL	decreased	
IgA	168 mg/dL	93–393 mg/dL	normal	
IgM	37 mg/dL	33–183 mg/dL	normal	
ANA	<1:40	<1:40	normal	
AMA	negative	negative	normal	
M-protein	negative	negative	normal	
Kappa light chain	600.4 mg/L	3.3–19.4 mg/L	elevated	
Lambda light chain	11.3 mg/L	5.7–26.3 mg/L	normal	
Kappa–Lambda ratio	53.13	0.26–1.65	elevated	
BNP	83.3 pg/mL	<18.4 pg/mL	elevated	
TSH	13.66 μU/mL	0.61–4.23μU/mL	elevated	
FT3	1.6 pg/mL	1.91–3.01 pg/mL	decreased	
FT4	0.8 ng/dL	0.83–1.53 pg/mL	decreased	
TPOAb	<9.0 U/mL	<16 U/mlL	normal	
TgAb	10.6 U/mL	<28 U/mL	normal	
Tg	153 ng/mL	<33.7 ng/mL	elevated	
TRAb	<0.8 U/L	<0.8 U/L	normal	
HBs antigen	negative	negative	normal	
HCV antibody	negative	negative	normal	
Urinary test				
Urinary RBC	2.4/HPF	<4/HPF	normal	
Urinary protein	5.55 g/gCr	<0.15 g/gCr	elevated	
Urinary protein (24-h collection)	6885 mg/day	<120 mg/day	elevated	
BJP	negative	negative	normal	

Kidney biopsy findings

**Figure 2:**
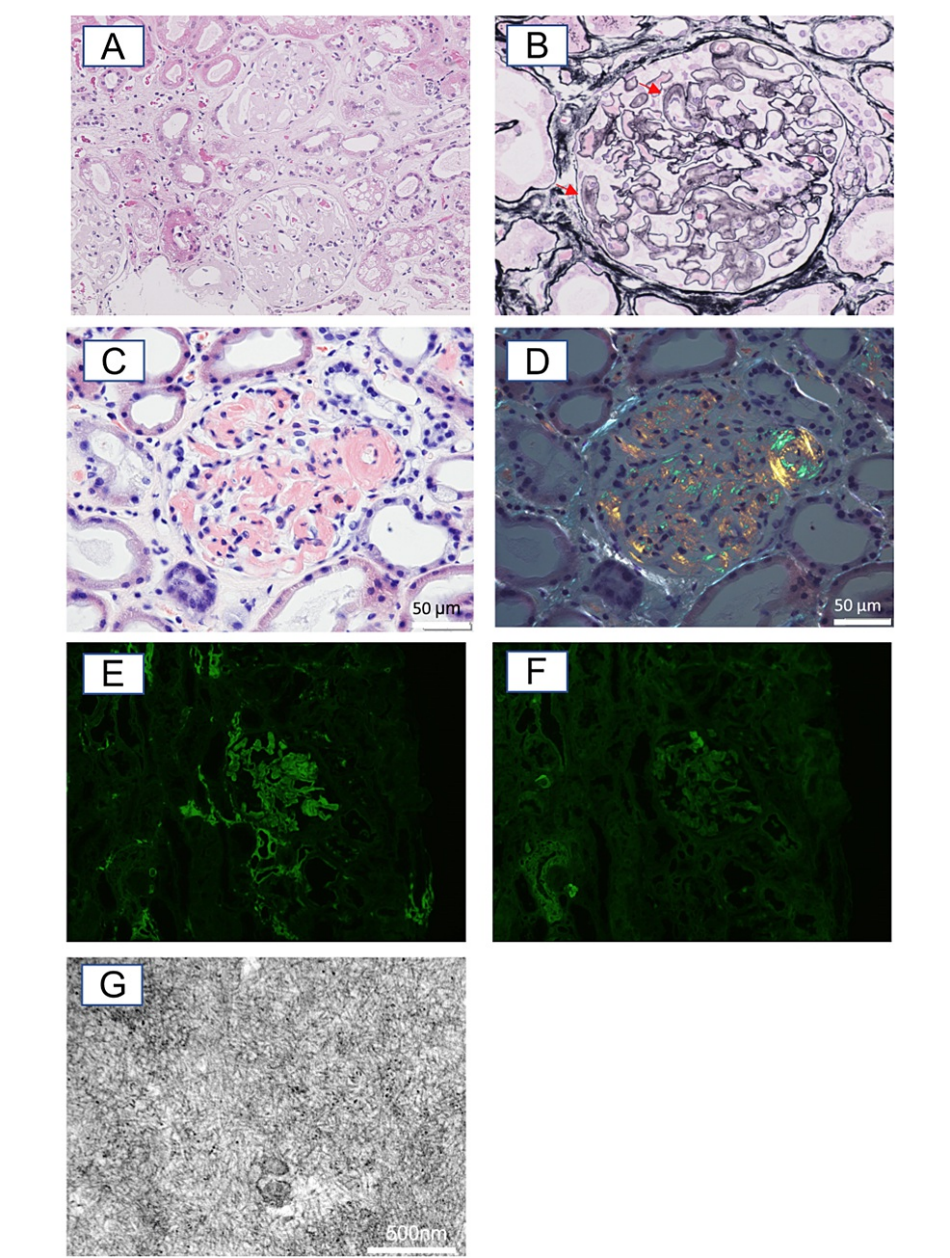
Pathological findings in the kidney (a) Capillary wall thickness observed in optical microscopy (hematoxylin-eosin staining; magnification, ×200). (b) Silver-positive spicules in the glomerular basement membrane (GBM) (red arrows) and depositions in the mesangium, peripheral capillary loops, and arterioles (periodic acid–methenamine silver staining; magnification, ×400). (c-d) Congo red stain positive deposition (c) with apple-green birefringence under polarized light (d). (e-f) Predominant kappa light chain (e) compared to lambda light chain (f) in Immunofluorescence studies. (g) Nonbranching fibrils with a diameter of about 10 nm in GBM (Electron microscopy, magnification, ×30000).

He was referred to the nephrology department due to nephrotic syndrome, and a renal biopsy was subsequently performed, which showed 30 glomeruli; one had glomerular sclerosis. Light microscopic analyses revealed a thickened capillary wall (Figure 2A), silver-positive spicules in the glomerular basement membrane (GBM), and amorphous depositions in the mesangium, peripheral capillary loops, and arterioles (Figure 2B). Congo red staining was positive with apple-green birefringence under polarized light (Figures 2C-2D). Immunofluorescence studies showed kappa light chain staining that was restricted to the GBM (Figures 2E-2F). Electron microscopy revealed nonbranching fibrils, with a diameter of 10 nm, in the GBM (Figure 2G).

Bone marrow examination and mass septectomy

Additional tests for myeloma were performed. Bone marrow examination showed a normocellular marrow with a mild increase in plasma cells. In situ hybridization showed kappa-positive cells without lambda-positive cells. Myeloma protein was not detected in the serum, but an abnormal kappa/lambda light chain ratio was noted as shown in Table 2 and the kidney tissue (Figure 2) showed amyloid depositions. For confirmation, we performed a mass septectomy, which revealed immunoglobulin kappa light chains. Hence, he was diagnosed as having AL amyloidosis. Although we considered initiation of treatment, unfortunately, he died five days after renal biopsy because of liver failure.

## Discussion

We reported a case of systemic AL amyloidosis with cholestatic liver failure, which is a rare but severe complication [4]. Despite improvements in treatment and prognosis [5], in our case, rapid disease progression hindered effective treatment initiation. However, because systemic AL amyloidosis presents with a heterogeneous clinical picture depending on the organ involved [2], it is difficult to diagnose the disease in its early stages. Given that previous studies suggested that cases with cholestatic liver involvement are associated with rapid disease progression similar to our case [3,4,6], early diagnosis is crucial for early treatment initiation. Unfortunately, there is no established marker for the early diagnosis of the disease. Although we did not perform the liver biopsy to determine amyloid deposition in the liver tissue, our case suggests that new-onset hypercholesterolemia with elevated GGT levels may be an early sign of AL amyloidosis with cholestatic liver involvement.

Amyloid deposition in the liver is common in systemic AL amyloidosis [7]; however, liver involvement rarely causes clinical manifestations [7,8]. Cases with liver involvement manifest with hepatomegaly and elevated ALP levels in 14%-30% of patients [2,4]. Previous studies showed that patients with biopsy-proven liver involvement with AL amyloidosis have poor prognosis [7,8]. Furthermore, rapidly progressive cases of AL amyloidosis with cholestatic liver failure leading to death have been reported [3,4,6]. Consistent with our case, several studies suggested that elevated total bilirubin levels are associated with short survival [7-9]. As Gertz and Kyle speculated, increased levels of total bilirubin are a sign of preterminal disease [8], similar to our case, where total bilirubin levels increased after hypercholesterolemia and elevated GGT levels were noted. Zhang et al. reported elevated GGT levels in 88.2% of patients with liver involvement due to AL amyloidosis [9]. Park et al. reported that 80% of patients with hepatic AL amyloidosis had hypercholesterolemia. These studies suggest that elevated GGT levels and hypercholesterolemia may be associated with liver involvement due to AL amyloidosis. Nevertheless, the clinical relevance of elevated GGT levels and hypercholesterolemia in AL amyloidosis remains unknown.

Few reports have focused on GGT and hypercholesterolemia independently as early markers in AL amyloidosis. Takao et al. reported that mild elevation of GGT levels could be an early marker of AL amyloidosis with liver failure [6]. Couture et al. speculated that new-onset hypercholesterolemia may be a manifestation of AL amyloidosis [10]. They found that levels of LDL-cholesterol are predominantly elevated in patients with hepatic AL amyloidosis. Takao et al. did not report cholesterol levels, whereas Couture et al. did not report GGT levels but both their reports support the findings in our case.

GGT is located in the plasma membranes of hepatocytes and is a biomarker of cholestatic liver failure [11]. However, GGT levels could be elevated in common diseases such as gastrointestinal disorders, pancreatitis, myocardial infection, diabetes mellitus, obesity, hyperthyroidism, lung disease, neurologic disease, rheumatic disease, infections, renal insufficiency, kidney transplantation, and alcoholism [11]. Hypercholesterolemia is also common since its estimated global prevalence is 11.9%-39% [12]. In addition to its high prevalence, hypercholesterolemia could be present in smokers and in those with hypothyroidism, diabetes mellitus, nephrotic syndrome, and alcoholism [13]. Primary biliary cholangitis (PBC) may also be considered as a differential diagnosis in cases of elevated GGT and cholesterol levels [14]. PBC causes T-cell-mediated destruction of intrahepatic bile ducts [15]. Although its cause remains unknown, AMA is a specific marker of PBC [15]. Slow progression of cholestasis followed by hepatic dysfunction is the common clinical picture of patients with PBC [15]. In addition to AMA, GGT and ALP are biomarkers of PBC [15], whereas hypercholesterolemia presents in 75%-95% of patients with the disease [14]. Reduced bile acid secretion and decreased lecithin-cholesterol acyltransferase lead to hypercholesterolemia in PBC [14]. Considering amyloidosis based only on elevated levels of GGT or hypercholesterolemia is difficult because they can be manifestations of other common diseases. However, if we focused on the combination of unexpected GGT and cholesterol-level elevation, AL amyloidosis could be considered a differential diagnosis when we exclude diabetes mellitus, alcoholism, and PBC.

Systemic AL amyloidosis may involve several organs. Cardiac involvement presents as restrictive cardiomyopathy with preserved ejection fraction [2]. As in our case, increased left ventricular thickening is a typical echocardiography finding [16]. Based on levels of N-terminal pro-B-type natriuretic peptide and cardiac biomarkers troponin-T, the Mayo Group proposed cardiac involvement staging in 2012 [17]. Renal involvement manifests with albuminuria, with approximately 40% of cases showing nephrotic-range albuminuria [2], and approximately 20% of cases progressing to end-stage renal insufficiency [2]. Hypothyroidism, which is a known independent negative prognostic factor in AL amyloidosis [18], is present in 19% of patients with AL amyloidosis [2]. Soft tissue involvement is seen in 17% of patients [2] and includes submandibular gland enlargement, macroglossia, symmetric cervical lymphadenopathy, and amyloid arthropathy [19]. Peripheral autonomous nerve involvement is seen in 12% and 10% of cases with AL amyloidosis, respectively [2]. Autonomous nerve involvement presents as orthostatic hypotension, resolution of pre-existing hypertension, erectile dysfunction, and either constipation or diarrhea [2]. Our case had multiorgan involvement in addition to cholestatic liver failure, including nephrotic syndrome (kidney involvement), left ventricular thickening (cardiac involvement), and hypothyroidism.

## Conclusions

In conclusion, the combination of elevated GGT levels and hypercholesterolemia may help diagnose hepatic AL amyloidosis, which, to the best of our knowledge, our report is the first to suggest. If patients manifest with progressive liver failure or other organ dysfunction, organ biopsy and serum kappa/lambda light chain testing should be considered. Further studies are warranted to confirm the preclinical characteristics of AL amyloidosis to diagnose hepatic AL amyloidosis for early treatment initiation.

## References

[1] Ryšavá R (2019). AL amyloidosis: advances in diagnostics and treatment. Nephrol Dial Transplant.

[2] Nuvolone M, Milani P, Palladini G, Merlini G (2018). Management of the elderly patient with AL amyloidosis. Eur J Intern Med.

[3] Peters RA, Koukoulis G, Gimson A, Portmann B, Westaby D, Williams R (1994). Primary amyloidosis and severe intrahepatic cholestatic jaundice. Gut.

[4] Desport E, Bridoux F, Sirac C (2012). Al amyloidosis. Orphanet J Rare Dis.

[5] Staron A, Zheng L, Doros G, Connors LH, Mendelson LM, Joshi T, Sanchorawala V (2021). Marked progress in AL amyloidosis survival: a 40-year longitudinal natural history study. Blood Cancer J.

[6] Takao S, Tanaka K, Miyazaki M (2013). A case of fatal intrahepatic cholestasis with primary AL amyloidosis: is early diagnosis possible?. Clin J Gastroenterol.

[7] Park MA, Mueller PS, Kyle RA, Larson DR, Plevak MF, Gertz MA (2003). Primary (AL) hepatic amyloidosis: clinical features and natural history in 98 patients. Medicine (Baltimore).

[8] Gertz MA, Kyle RA (1988). Hepatic amyloidosis (Primary [AL], immunoglobulin light chain): the natural history in 80 patients. Am J Med.

[9] Zhang LL, Shen KN, Zhang CL (2019). Clinical presentation and prognostic analysis of Chinese patients with systemic light chain amyloidosis with liver involvement. Leuk Res.

[10] Couture P, LeBlanc F, Gagnon P, Gagnon O, Gagné C (1997). Hyperlipidemia as the first biochemical manifestation of primary hepatic amyloidosis. Am J Gastroenterol.

[11] Pollock G, Minuk GY (2017). Diagnostic considerations for cholestatic liver disease. J Gastroenterol Hepatol.

[12] Al-Zahrani J, Shubair MM, Al-Ghamdi S (2021). The prevalence of hypercholesterolemia and associated risk factors in Al-Kharj population, Saudi Arabia: a cross-sectional survey. BMC Cardiovasc Disord.

[13] Ibrahim MA, Asuka E, Jialal I (2022). Hypercholesterolemia. https://www.ncbi.nlm.nih.gov/books/NBK459188/.

[14] Wah-Suarez MI, Danford CJ, Patwardhan VR, Jiang ZG, Bonder A (2019). Hyperlipidaemia in primary biliary cholangitis: treatment, safety and efficacy. Frontline Gastroenterol.

[15] Younossi ZM, Bernstein D, Shiffman ML, Kwo P, Kim WR, Kowdley KV, Jacobson IM (2019). Diagnosis and management of primary biliary cholangitis. Am J Gastroenterol.

[16] Stern LK, Kittleson MM (2021). Updates in cardiac amyloidosis diagnosis and treatment. Curr Oncol Rep.

[17] Kumar S, Dispenzieri A, Lacy MQ (2012). Revised prognostic staging system for light chain amyloidosis incorporating cardiac biomarkers and serum free light chain measurements. J Clin Oncol.

[18] Muchtar E, Dean DS, Dispenzieri A (2017). Prevalence and predictors of thyroid functional abnormalities in newly diagnosed AL amyloidosis. J Intern Med.

[19] Prokaeva T, Spencer B, Kaut M (2007). Soft tissue, joint, and bone manifestations of AL amyloidosis: clinical presentation, molecular features, and survival. Arthritis Rheum.

